# Evaluation of thymus morphology and serum cortisol concentration as indirect biomarkers to detect low-dose dexamethasone illegal treatment in beef cattle

**DOI:** 10.1186/1746-6148-8-129

**Published:** 2012-08-03

**Authors:** Marta Vascellari, Katia Capello, Annalisa Stefani, Giancarlo Biancotto, Letizia Moro, Roberto Stella, Giandomenico Pozza, Franco Mutinelli

**Affiliations:** 1 Istituto Zooprofilattico Sperimentale delle Venezie, viale dell’Università 10, 35020, Legnaro, PD, Italy

**Keywords:** Beef cattle, Indirect biomarker, Cortisol, Cortex/medulla ratio, Dexamethasone, Thymus

## Abstract

**Background:**

Corticosteroids are illegally used in several countries as growth promoters in veal calves and beef cattle, either alone or in association with sex steroids and β-agonists, especially at low dosages and primarily through oral administration, in order to enhance carcasses and meat quality traits. The aim of the present study is to evaluate the reliability of the histological evaluation of the thymus, as well as the serum cortisol determination, in identifying beef cattle, treated with two different dexamethasone-based growth-promoting protocols and the application of different withdrawal times before slaughter.

**Results:**

Our findings demonstrate that low dosages of dexamethasone (DXM), administered alone or in association with clenbuterol as growth promoter in beef cattle, induce morphologic changes in the thymus, resulting in increase fat infiltration with concurrent cortical atrophy and reduction of the cortex/medulla ratio (C/M). In fact, the C/M value was significantly lower in treated animals than in control ones, with both the protocols applied. The cut off value of 0.93 for the cortex/medulla ratio resulted to be highly effective to distinguish control and treated animals. The animals treated with DXM showed inhibition of cortisol secretion during the treatment period, as well as at the slaughterhouse, 3 days after treatment suspension. The animals treated with lower doses of DXM in association with clenbuterol, showed inhibition of cortisol secretion during the treatment period, but serum cortisol concentration was restored to physiological levels at slaughterhouse, 8 days after treatment suspension.

**Conclusions:**

The histological evaluation of thymus morphology, and particularly of the C/M may represent a valuable and reproducible method applicable to large-scale screening programs, due to the easy sampling procedures at slaughterhouse, as well as time and cost-saving of the analysis. Serum cortisol determination could be considered as an useful *in vivo* biomarker of dexamethasone illegal treatment in beef cattle during the fattening period, whilst it does not appear to be a good biomarker at the slaughterhouse, since the protocol of DXM administration, as well as the withdrawal period could affect the reliability of the method.

## Background

Corticosteroids are illegally used in several countries as growth promoters in veal calves and beef cattle, either alone or in combination with anabolic agents, especially at low dosages and primarily through oral administration, in order to enhance carcasses and meat quality traits [1,2]. These drugs are usually used in association with sex steroids and β-agonists to obtain a higher proportion of lean meat [3]. The strong pharmacological activity of synthetic corticosteroids renders the residues of these molecules potentially dangerous for meat consumers. As a consequence, corticosteroids administration as growth-promoters is banned in the EU (Council Directive 96/23/EC) [4]. For therapeutic indications only, the use of some glucocorticosteroids is allowed and therefore maximum residue limits (MRLs) have been established for bovine edible tissues (0.75 μg/kg in kidney and muscle, and 2 μg/kg in liver) and milk (0.3 μg/kg) (Commission Regulation (EC) N. 508/1999) [5].

Unfortunately, the targeted gas chromatography/mass spectrometry (GC/MS) and liquid chromatography tandem mass spectrometry (LC/MS/MS) methods used may give unsatisfactory results, due to the growing use of very low individual doses, achieved by combining several products to produce synergistic effects (‘cocktails’), as well as the use of products known to be difficult to analyze because of their rapid metabolism [6]. Furthermore, the use of low-dosage drug cocktails causes only minimal tissue changes thus hampering the detection of suspected alterations in target organs [6,7]. For these reasons, screening methods based on different approaches, including the measurement of indirect biomarkers and the use of histological and physiological indicators, are strongly required. Innovative methods that can be useful in identifying animals treated with anabolic agents include the so-called omics techniques (transcriptomic, proteomic and metabolomic), based on the simultaneous detection of biomarkers predictive of administration of specific substances [8-12]. Since 2009, histological examination of the thymus has been introduced by the Ministry of Health in Italy as a screening test to control the illegal use of corticosteroids in veal calves and beef cattle (Piano Nazionale Residui 2009 – Ministero della Salute) [13]. The role of histological examination of the thymus as a biomarker of illegal corticosteroid treatment has been established [7,14,15], but it is nevertheless difficult to discriminate between anabolic and therapeutic treatment. In addition, in adult cattle, it is difficult to distinguish between the physiological rate of involution and the atrophy induced by treatment.

If the effect of non-therapeutic doses of dexamethasone in beef cattle on growth performance and carcass traits is well known [1,2,6], its effect on hormonal, hematological and biochemical parameters is still matter of investigation. The potential modification of hematological and endocrine parameters induced by treatment could be used to identify illegally treated animals at the farm and slaughterhouse levels, and the association of additional indirect indicators may be helpful in enhancing the sensitivity and specificity of the method.

The aim of the present study was to evaluate the reliability of the histological evaluation of the thymus, as well as the serum cortisol determination, in identifying beef cattle treated with two different dexamethasone-based growth-promoting protocols and the application of different withdrawal times before slaughter.

## Methods

### Animals and treatments

A total of 42 Charolaise beef cattle, aged between 19 and 21 months, were bought by local farms and underwent clinical controls by a veterinarian. All the animals were vaccinated against IBR, Para Influenza (PI3), BRSV and BVDV (CATTLEMASTER® 4; Pfizer Animal Health, New York, USA) and treated against parasites with ivermectina (Ivomec®, Merial, Italy). After a 19 days of acclimation period the animals were randomly divided in 3 groups: group A (n = 18) that served as control group; in group B (n = 16) 0.75 mg/day dexamethasone-21-sodium-phosphate (Desashock®; Fort Dodge Veterinaria, Olot, Spain) were orally administered for 42 days (days 1–42); group C (n = 8) was given 0.66 mg/day dexamethasone-21-sodium-phosphate (Desashock®; Fort Dodge Veterinaria, Olot, Spain) for 21 days (days 8–28) and clenbuterol hydrochloride (Sigma Aldrich, St. Louis, MO) at doses of 2 mg/day (days 1–7), 4 mg/day (days 8–14) and 6 mg/day (days 15-28) orally. Animals of group A and B were slaughtered after a three-day withdrawal period, while the animals of group C were slaughtered after a eight-day withdrawal period. All the animals were kept in separate boxes, and fed with a diet consisting of hay and a commercial compound feed based on hybrid corn, soybean meal, bran and barley; water was supplied ad libitum. Clinical check-ups were carried out daily by a veterinarian. At the end of the sampling procedure carcasses of treated animals were destroyed according to the law in force.

The experiment was authorised by the Italian Ministry of Health and the Ethics Committee of the University of Bologna and carried out according to European Economic Community Council Directive 86/609 [16], which is recognized and adopted by the Italian Government (D.L. 27/01/1992 no. 116) [17].

### Chemical analysis

Drug administration during the whole treatment protocol was monitored collecting urine in the morning, once a week. The residual levels of dexamethasone (DXM) in urine and liver at slaughterhouse were also determined. DXM in urine and liver was calculated by means of internally validated analytical methods. Briefly, urine samples, after deconjugation with helix pomatia, were subjected to a purification step by solid phase extraction (SPE) on a C18 column (500 mg/6 mL) followed by a filtration through a NH_2_ SPE cartridge (100 mg, 1 mL), while liver samples were extracted with acetonitrile and purified by a single C18 (500 mg/6 mL) SPE step. Purified extracts were finally analyzed by an HPLC (Alliance 2695, Milford, MA, USA, Waters), coupled to a triple-quadrupole mass spectrometer (Quattro Ultima, Micromass, Waters, Milford, MA, USA) operating in negative electrospray ionization. The chromatographic separation was achieved by a Synergi-polar C18 column (150 x 2.00 mm, 4 μm, Phenomenex, Torrance, CA, USA), adopting 0.05% (v/v) formic acid in water (eluent A) and 0.05% (v/v) formic acid in acetonitrile (eluent B) as mobile phases. Gradient elution was performed combining the mobile phases (A:B) as follows: 85:15 from 0 to 1 min, 70:30 at 5 min, 70:30 at 6 min, 65:35 at 16 min, 20:80 from 17 to 20 min, and then 85:15 from 21 to 26 min to re-equilibrate the system. The flow-rate was set to 0.250 mL min^-1^ and the injection volume was 10 μL.

Mass spectrometric analyses were performed using the following settings: nebulisation and desolvation were achieved using nitrogen gas at flow-rates of 50 and 900 L h^-1^, respectively. Source and desolvation temperatures were 125°C and 325°C. The potential applied on the capillary was 2.7 kV and the potential applied on the cone was optimized for each molecule in the range between 40–50 V. MS/MS experiments were performed using argon as the collision gas at a pressure of 3.5*10^-3^ mbar and two selective transitions (437 > 361 m/z and at 437 > 307 m/z, collision energy equal to 18 and 31 eV respectively) were chosen for unequivocal identification of DXM residues. DXM was identified on the basis of the retention time and ion ratios of the selected transitions. Quantification was performed adopting dexamethasone-D4 (deuterated DXM, 441 > 363 m/z, 19 eV) as internal standard.

The methods were developed and validated according to the guidelines laid down by 2002/657/EC Commission Decision [18]. Specificity was assessed on twenty different blank samples (i.e. bovine urine and liver) that were extracted and analyzed to verify the absence of interferences due to the matrix or endogenous components. The linearity of the LC-MS/MS response was verified using four calibration levels of standard solutions in matrix to take into account the matrix effect on ionization. Recovery and repeatability were assayed in three separate working sessions, by spiking blank samples at three different concentrations with six replicates for each level. Quantification was achieved by internal standard method using independent calibration curves in each session. Decision limits were calculated according to the calibration curve procedure established by ISO 11843.

### Histopathology

Portions of the thoracic thymus of each animal were collected soon after slaughter and fixed in 10% neutral buffered formalin and paraffin embedded according to routine procedures. Representative sections of each sample were stained with hematoxylin and eosin for histological examination. Histopathological examinations were blindly performed. The morphology of the thymus parenchyma was evaluated for adipose tissue infiltration, with concurrent cortical atrophy, and a score from 1 to 3 (mild, moderate, severe) was attributed.

**Figure 1:**
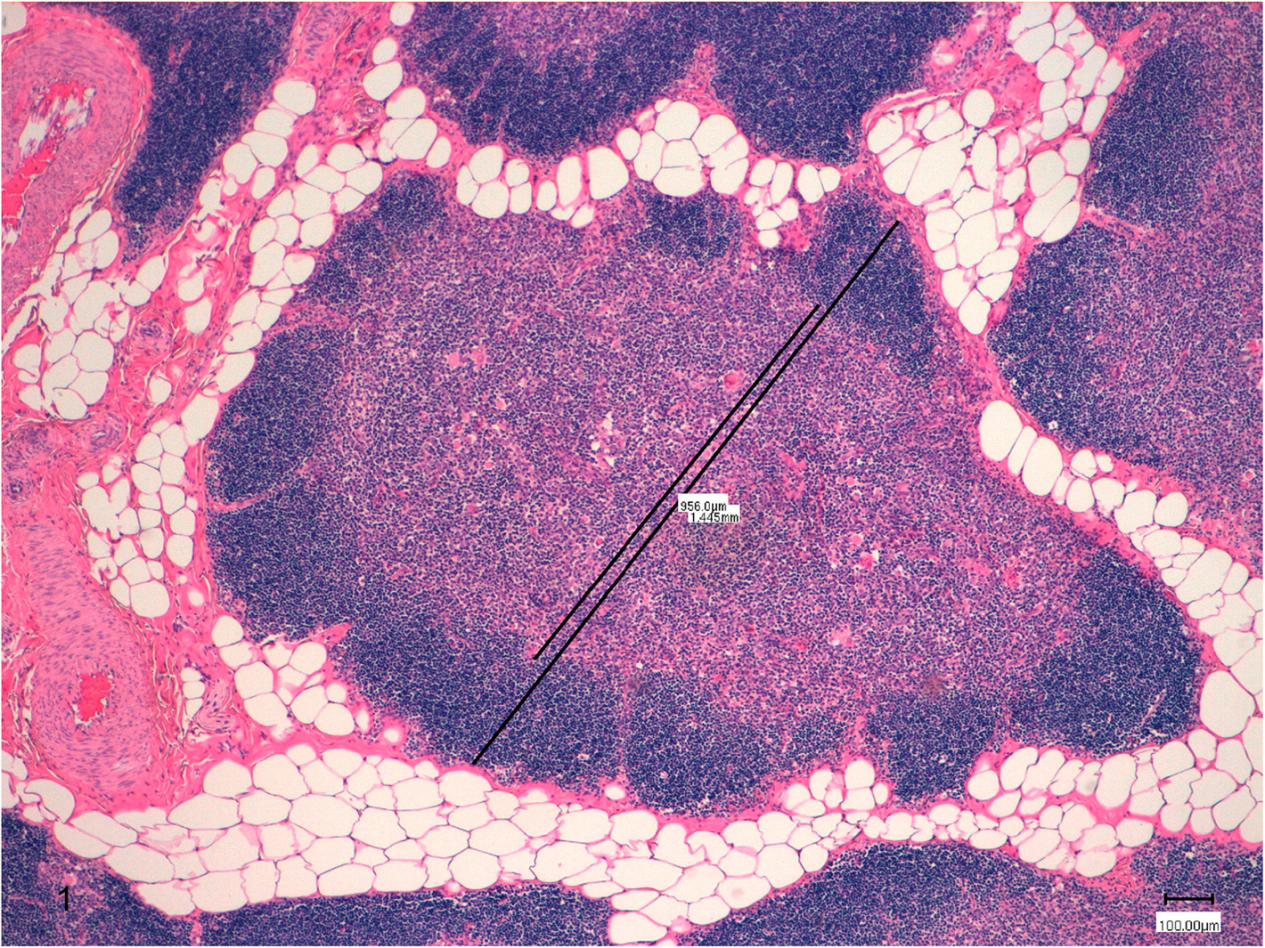
**Measurement of the thymus cortex and medulla.** Extension of the cortex was measured against a graduated line, starting and ending at the interlobular connective or adipose tissue; a second parallel line was drawn to measure medulla thickness (HE 5X).

Furthermore, the thymus sections were examined at low magnification (4x) using a digital microimaging device (Leica DMD108 Digital microimaging device for clinical diagnostics labs, Wetzlar, Germany) to evaluate cortex and medulla thickness, according to the method described by Bozzetta et al. [19]. For each slide, 5 functional lobules, composed of an outer cortex and inner medulla and surrounded by connective and adipose tissues, were randomly selected and the extension of the cortex and medulla were measured against a graduated line, starting and ending at the interlobular connective or adipose tissue; a second parallel line was drawn to measure medulla thickness (Figure 1). The cortex thickness was obtained by subtracting the second value from the first one and the cortex/medulla ratio (C/M) was calculated.

### Serum cortisol determination

Individual blood samples were taken from the jugular vein of the animals of the three groups, in the morning of the first day of treatment, before drugs administration (T0), on day 28 of treatment (T28) and at slaughterhouse, before stunning. Blood was collected in 10 mL tubes without anticoagulant (Becton Dickinson, Meylan Cedex, France) in the morning, before diet administration and at slaughterhouse immediately after stunning. Serum samples were obtained after clot formation at room temperature and centrifugation at 2500 g at 20°C for 10 minutes. Serum cortisol concentration was determined using a commercial kit (Catalog Number: LKCO1, Siemens Medical Solutions Diagnostics, Flanders, NJ), performed with the automated analyzer Immulite One® (Siemens Medical Solutions Diagnostics, Flanders, NJ) by chemiluminescent immunometric assays. The method underwent internal validation. Analytical variability was measured with the use of two internal serum pools with low (7,4 nmol/L) and high (87.9 nmol/L) cortisol concentration; intra and inter-assay coefficients of variation were 12.9% and 14.0% for low serum pool, 5.1% and 7.4% for high serum pool. The analytical sensitivity of the assay was 5.5 nmol/L. The cortisol serum concentration values under the analytical sensitivity were considered equal to 5.5 nmol/L.

### Statistical analysis

The association between thymus score and group was assessed by Fisher’s exact test.

The distributions of cortex thickness, medulla thickness and C/M among groups were evaluated using a linear mixed model, after having transformed the data using square root function for the first two parameters and logarithmic function for the last one, in order to fit the Gaussian distribution of data. The fixed effect was the treatment group while the random effect was the animal nested within group; lobules, collected for each animal, represented the residual. The analysis was performed considering the three groups (A, B and C) and B and C together (treated group). In the former, all pairwise comparisons were carried out using Bonferroni adjustment. Furthermore, the linear mixed model, stratified by treatment group, was used to compare the distribution of C/M among thymus scores (fixed effect). The analysis of residuals was adopted for checking the models.

With regard to animals with thymus score 2, a ROC analysis was carried out to identify a cut-off of the C/M able to discriminate lobules belonging to treated and untreated animals; for this analysis the five lobules, for each thymus sample, were considered as independent statistical units and, given that lower values of C/M were supposed to belong to treated animals, the ratio was multiplied by −1. To discriminate treated and untreated animals, the number of lobules for each thymus sample with a C/M under the cut-off value was calculated, and positive and negative predictive values (PPV and NPV, respectively) were provided considering the treatment group as gold standard.

The serum cortisol concentrations of the three treatment groups, stratified by sampling time, were evaluated using the Kruskal-Wallis test. The Wilcoxon rank-sum test was used for the pairwise comparisons and the Bonferroni-corrected alpha was used to evaluate the results. In addition, robust test for equality of variance, with the Brown and Forsythe adjustment, compared the variability of the distributions across the groups. For each treatment group, the serum cortisol concentration among different sampling times was evaluated by the Wilcoxon matched-pairs signed-ranks test.

All data analyses were performed using SAS 9.1 Version (SAS Institutem Inc., Cary, NC, USA).

The P value of 0.05 was considered as the level of statistical significance.

## Results

All the farmed animals concluded the experimental trial without insurgence of clinical complications and therefore no medical treatments were required.

### Chemical analysis

The analytical methods fulfilled the European requirements and were considered adequate for the analytical purposes of this study; in particular, recovery was better than 95%, precision was lower than 6.5% and calculated decision limits (CCα) were 0.251 μg/L in urines, and 0.247 μg/Kg in liver.

Analysis of urine samples collected during the treatment period demonstrated that the oral administration protocol resulted in uptake of DXM (average DXM ranged between 0.8 and 2.2 μg/L) in both groups B and C.

In group B (3-days withdrawal period) DXM urinary concentration at the slaughterhouse ranged from 0 to 0.48 μg/L. Only 7 out of 16 animals (43.8%) showed a DXM residue higher than the CCα of the method (0.251 μg/L). DXM residues in liver samples (>CCα) were detected in all the animals of group B, with concentrations ranging between 0.70 and 7.40 μg/Kg.

In group C (8-day withdrawal period) all the urine samples collected at the slaughterhouse had DXM concentration largely below the CCα, with putative extrapolated values ranging between 0 and 0.03 μg/L; by contrast in liver samples DXM residues (>CCα) were detected in 2 out of 8 animals (25%).

As expected, no DXM residues were detected in urine and liver samples collected during the treatment period as well as at the slaughterhouse, from animals of the control group A. Table 1 reports the mean values ± standard deviations calculated taking into account only samples with residual concentrations higher than CCα.

**Table 1:** Chemical analysis of DXM residues (>CCα) from liver and urine samples collected at the slaughterhouse

**Sample**	**Number of positive animals (mean value ± Standard deviation)**		
	**Group A**	**Group B**	**Group C**
Liver [μg/kg]	0/18	16/16 (2.35 ± 1.81)	2/8 (0.28 ± 0.03)
Urine [μg/L]	0/18	7/16 (0.33 ± 0.09)	0/8

### Histopathology

**Figure 2:**
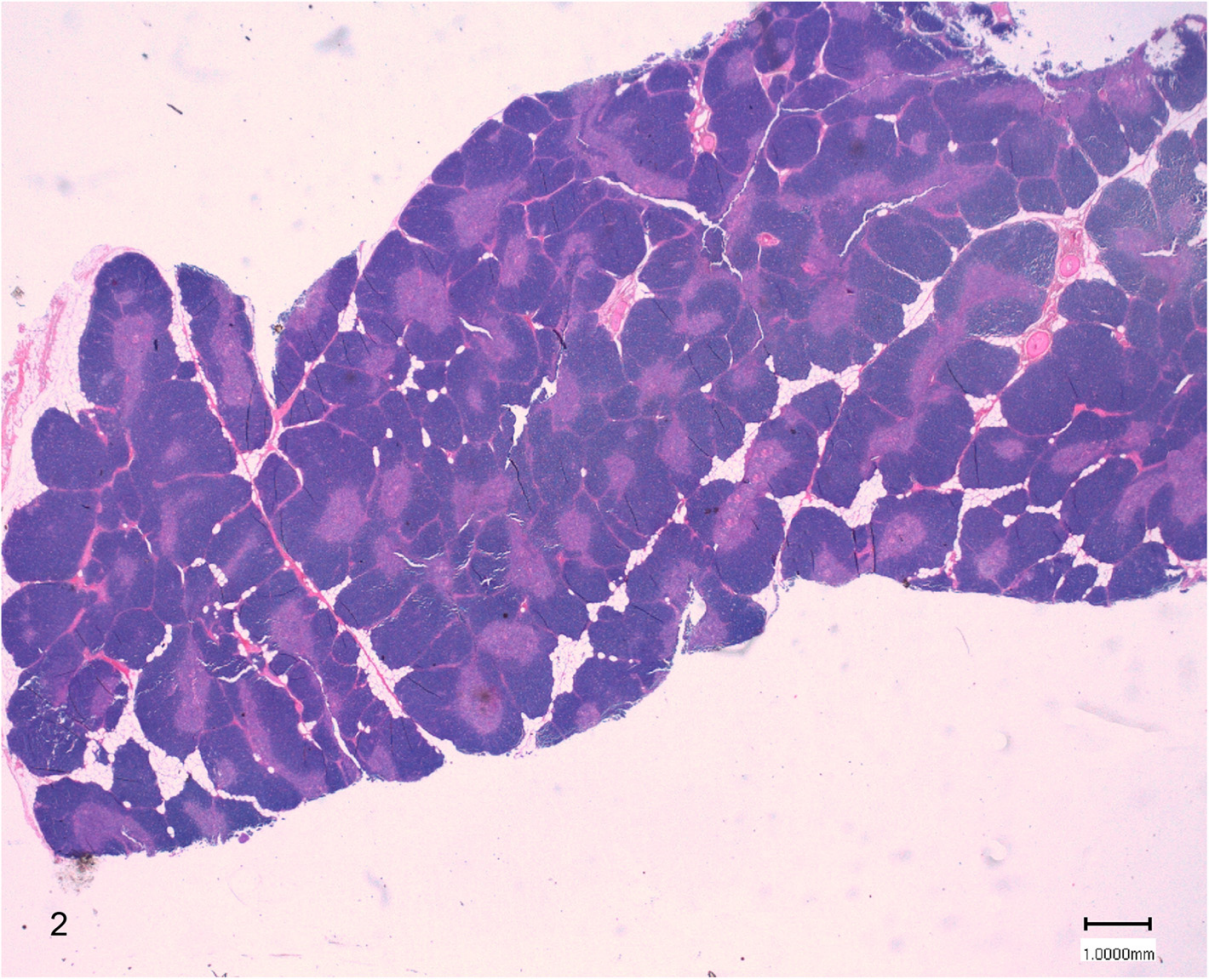
**Thymus morphology of a control animal (group A).** The score 1 was attributed to samples with slight physiological thymus atrophy, due to mild infiltration of adipose tissue at the periphery of the lobules (HE 0.3X).

**Figure 3:**
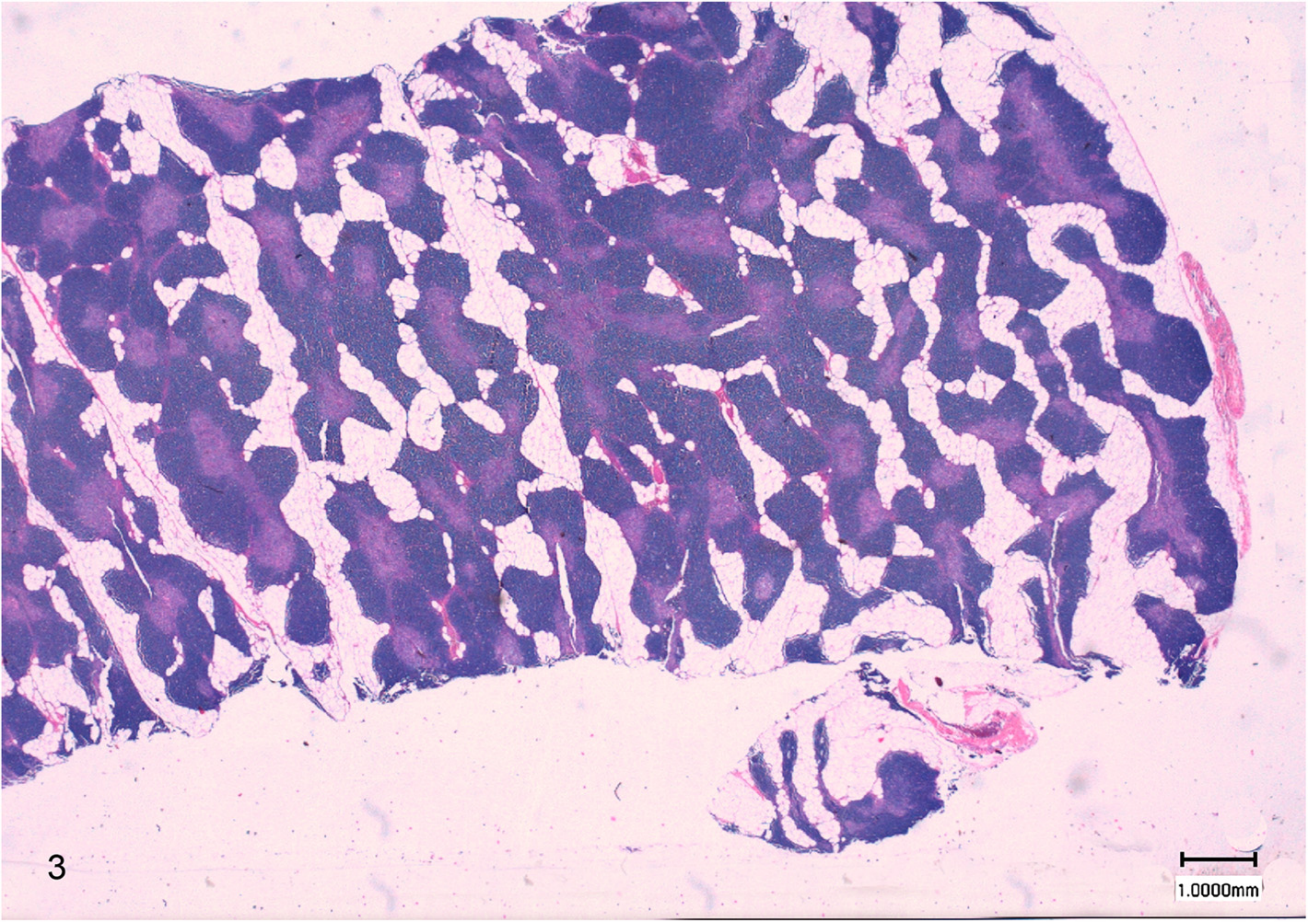
**Thymus morphology of a treated animal (group C).** The score 2 was referred to samples with moderate fat interstitial infiltration, with thinning and initial replacement of the cortex (HE 0.3X).

**Figure 4:**
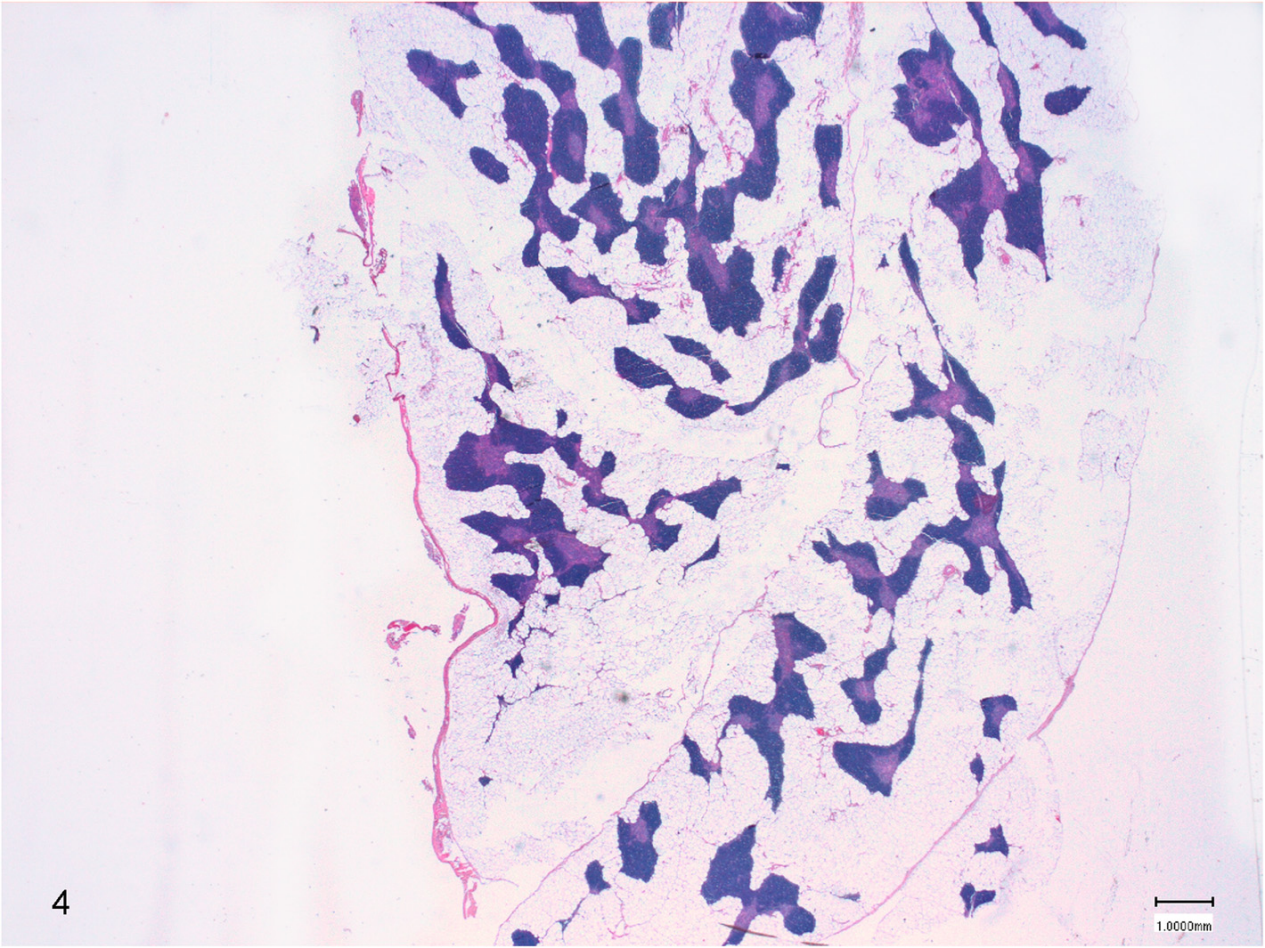
**Thymus morphology of a treated animal (group B).** the score 3 was attributed to samples with severe adipose tissue infiltration of the parenchyma, with pronounced cortex atrophy (HE 0.3X).

On histological examination, score 1 was attributed to samples with slight physiological thymus atrophy, due to mild infiltration of adipose tissue at the periphery of the lobules (Figure 2). Score 2 was referred to samples with moderate fat interstitial infiltration, with thinning and initial replacement of the cortex (Figure 3). Score 3 was attributed to samples with severe adipose tissue infiltration of the parenchyma, with pronounced cortex atrophy (Figure 4). In some samples the lobular structures were not recognizable, and they were almost completely replaced by fat tissue leaving only scattered lymphocytes aggregates. On blinded analysis, the scores 1 (n = 12) and 2 (n = 6), indicating the presence of mild and moderate amounts of infiltrating fat, respectively, were attributed to the group A (controls); in group B 4 samples were scored 2 and 12 samples were scored 3 (severe infiltration). In group C 6 samples were scored 2 and 2 were scored 3 (Table 2).

**Table 2:** Thymus scores distribution in different groups, based on the rate of adipose tissue infiltration, with concurrent thymus cortical atrophy

**Group**	**Thymus Score**	**N. of animals**	**%**
A	1	12	67
	2	6	33
	3	0	0
B	1	0	0
	2	4	25
	3	12	75
C	1	0	0
	2	6	75
	3	2	25

The thymus scores distributions resulted to be significantly different among the three groups (*P* < 0.001). When groups B and C (treated) were taken together the thymus scores were significantly associated with the steroidal treatment (*P* < 0.001).

**Figure 5:**
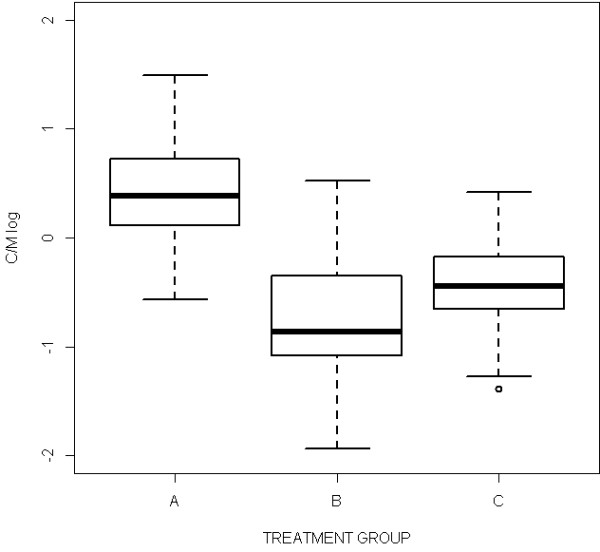
**The distribution of C/M values among the three groups.** The C/M values resulted significantly different between group A and B (*P* < 0.001), as well as between group A and C (*P* < 0.001), while no statistical differences were observed between groups B and C (*P* = 0.083).

**Figure 6:**
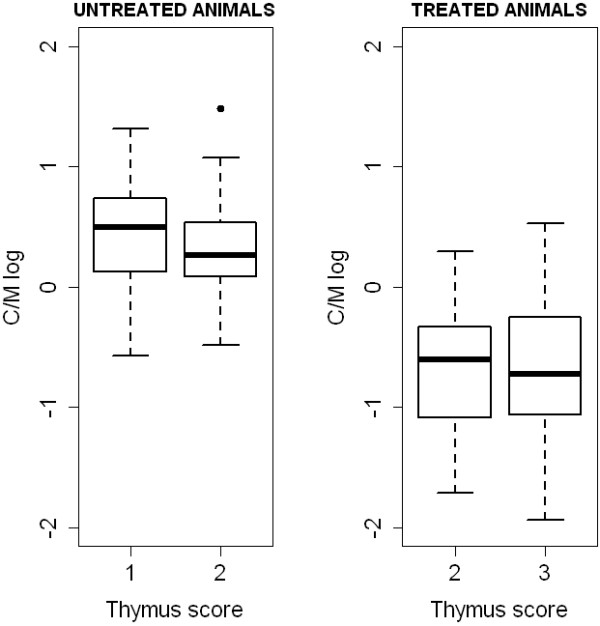
**Comparison of the C/M values of samples from the same treatment group, but with different thymus scores.** No significant differences were observed among control and treated animals with different thymus scores (group A: *P* = 0.414; group B + C: *P* =0.723).

Descriptive statistics for cortex thickness, medulla thickness and C/M, stratified by treatment group, are provided in Table 3. The cortex thickness resulted to be significantly different among groups (*P* < 0.001); on the contrary, the medulla thickness resulted to be not significantly different among groups (*P* 0.552). Considering the C/M, group A showed values ranging from 0.56 to 4.43 with an average of 1.62. In group B, the C/M was not calculated in 5 samples due to the severe fat infiltration and the lack of functional lobules; among the remaining samples, C/M values ranged from 0.14 to 1.69, with an average of 0.53. In group C the C/M was calculated for 7 out of 8 animals, and the values ranged from 0.25 to 1.51, with an average value of 0.69. Table 4 shows the results of the pairwise comparisons performed by linear mixed models. The distribution of C/M values among the three groups resulted to be significantly different (*P* < 0.001); particularly, the C/M values were significantly different between group A and B (*P* < 0.001), as well as between group A and C (*P* < 0.001), while no statistical differences were observed between groups B and C (*P* = 0.083) (Figure 5). Taking together groups B and C (treated) the C/M values resulted to be significantly different from those of the group A (*P* < 0.001). Comparing the C/M values of samples from the same treatment group, but with different thymus scores, no significant differences were observed (group A: *P* = 0.414; group B + C: *P* =0.723) (Figure 6).

**Table 3:** Descriptive statistics for cortex thickness (μm), medulla thickness (μm) and C/M of thymus samples, by treatment group

**Parameter**	**Group**	**N. of lobules**	**Mean**	**Median**	**SD**	**Min**	**Max**
Cortex thickness	A	90	795.4	763.5	254.4	298.0	1573.0
	B	55	230.7	205.0	110.7	76.0	650.0
	C	35	359.1	351.0	149.2	137.0	894.0
Medulla thickness	A	90	539.8	548.5	173.0	206.0	1044.0
	B	55	506.4	484.0	233.8	161.0	1120.0
	C	35	569.4	505.0	239.7	244.0	1573.0
C/M	A	90	1.62	1.47	0.71	0.56	4.43
	B	55	0.53	0.42	0.32	0.14	1.69
	C	35	0.69	0.64	0.31	0.25	1.51

**Table 4:** Results of linear mixed models applied to cortex thickness, medulla thickness and C/M ratio as dependent variables

**Dependent variable**	**Parameter**	**Estimate**	**Standard error**	**Adjusted p**
Cortex thickness square root	A vs B	13.0511	1.0086	<.0001
	A vs C	9.2794	1.1740	<.0001
	B vs C	−3.7717	1.2743	0.0170
Medulla thickness square root	A vs B	1.0167	1.1828	1.000
	A vs C	−0.5084	1.3766	1.000
	B vs C	−1.5251	1.4942	0.9445
C/M log	A vs B	1.1703	0.1055	<.0001
	A vs C	0.8634	0.1228	<.0001
	B vs C	−0.3070	0.1333	0.0833

**Figure 7:**
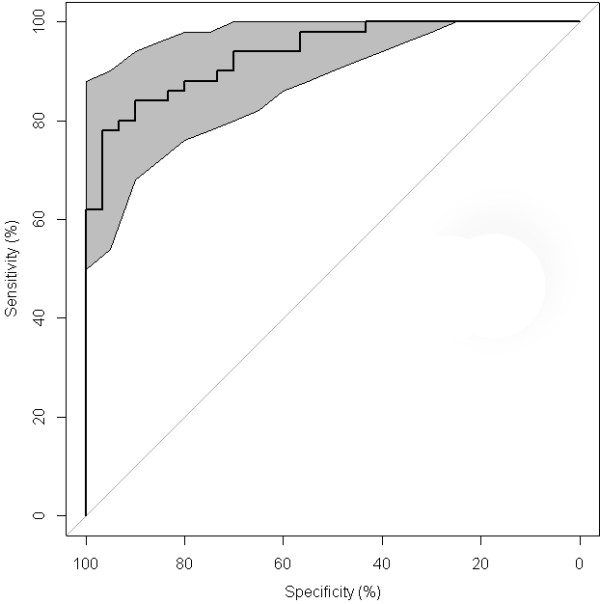
**ROC curve for C/M ratio with 95**% **confidence interval.** Taking in consideration the animals with thymus score 2, the best cut-off value of the C/M to discriminate treated and untreated samples resulted to be 0.93 (AUC 0.94; 95% CI 0.89-0.98).

Taking in consideration the animals with thymus score 2 (n = 15; 6 in group A, 4 in group B and 5 in group C), the distribution of the C/M values between the groups A and B, as well as A and C resulted to be significantly different (*P* <0.001), while no significant differences were observed between groups B and C (*P* = 0.200). Considering together the groups B and C (treated), the distribution of the C/M values were significantly different from the control group (*P* < 0.001). The best cut-off value of the C/M to discriminate treated and untreated samples resulted to be 0.93 (Area Under the Curve AUC 0.94; 95% CI 0.89-0.98) (Figure 7).

Considering five lobules for each thymus sample, animals with at least two lobules with C/M < 0.93 should be considered as treated, providing a PPV and NPV both equal to 100% (PPV 95%CI: 69.1%-100.0%; NPV 95%CI: 54.1%-100.0%).

### Serum cortisol concentration

Baseline T0 values of serum cortisol showed high variability in all the three groups, ranging from 5.5 nmol/l to 159.0 nmol/l , but no significant differences were found among the groups (*P* = 0.061) (Table 5).

**Table 5:** Descriptive statistics for serum cortisol concentration (nmol/L) by treatment groups at different sampling times

**Sampling time**	**Group**	**Mean**	**Median**	**St. dev.**	**Min**	**Max**
T0	A	48.2	43.3	34.2	5.7	108.0
	B	30.1	15.8	37.9	5.5	159.0
	C	24.3	11.8	26.0	5.5	76.1
T28	A	23.8	18.3	18.9	5.5	73.5
	B	5.9	5.5	1.5	5.5	11.6
	C	7.4	5.5	5.3	5.5	20.6
Slaughterhouse	A*	65.9	57.0	47.3	7.0	140.0
	B*§	9.1	5.5	7.8	5.5	35.4
	C**	82.3	86.8	25.5	34.8	116.0

* 3 days withdrawal period.

**8 days withdrawal period.

§one observation not considered because outlier.

At T28, 22 of 24 animals in groups B and C showed cortisol concentration under the analytical sensitivity and were considered equal to 5.5 nmol/L; the distribution of the parameter in group A had a mean and a median values of 23.8 nmol/L and 18.3 nmol/L, respectively, which resulted to significantly differ from the treated groups (*P* < 0.001).

At the slaughterhouse, no statistical differences were observed between the distributions of cortisol concentration of group A and group C (*P* = 0.317); but, a significant difference was found between group A and B (*P* < 0.001). For both comparisons, a significantly higher variability in group A than group C (*P* = 0.006) and group B (*P* < 0.001) was found. Different distribution (*P <* 0.001) of cortisol concentration but similar spread (*P* = 0.239) were highlighted between group B and C.

The analysis carried out comparing the three different sampling time in the control group (A) showed a significant decrease of the serum cortisol concentration at T28 compared to T0 (P 0.031), followed by a significant increase at the slaughterhouse (P 0.001); no differences were observed between T0 and slaughterhouse sampling (P 0.349).

Group B presented similar distributions of serum cortisol values at T28 and slaughterhouse (P 0.099), which were both significantly lower than at T0 (T28 vs T0: P 0.001; slaughterhouse vs T0: P 0.018).

With regard to group C, all pairwise comparisons showed that p-values were lower than 0.05 (T0 vs T28: P 0.019; T0 vs slaughterhouse: P 0.012; T28 vs slaughterhouse: P 0.012), with a significant decrease of the serum cortisol concentration during the treatment (T0 vs T28), followed by a significant increase at the slaughterhouse (T28 vs slaughterhouse and T0 vs slaughterhouse).

## Discussion

The assurance of safe and good quality food is a fundamental request of the consumers. Dexamethasone and other corticosteroids are frequently illegally used as growth-promoters in livestock production [6], often administered at low dosage alone or combined with β-agonists and/or anabolic steroids.

In our study, the results of the chemical analysis in urine and liver samples obtained at the slaughterhouse, confirmed that when DXM is administered at low dosage, its rapid metabolism and excretion makes the residues determination difficult, as the drug is readily depleted after the suspension of the treatment [20,21]. In fact, while in group B DXM residues were still detectable and identifiable in all liver samples, in animals of group C, which were treated with lower dosage of DXM and had a 8-day withdrawal period, DXM residues (>CCα) were not detectable in liver of 6 out of 8 animals; moreover, DXM residues detected in 2 liver samples were lower than the MRL = 2 μg/Kg in liver (Commission Regulation (EU) No 37/2010) [5]. Furthermore, the urine samples collected at the slaughterhouse, had DXM concentrations largely below the CCα in 9 animals of group B, as well as in all the animals of group C. These data are in accordance with the findings of a previously published paper [15] evaluating DXM urine excretion in beef cattle treated with low-dose of DXM during the drug administration period, as well as 6 days after the end of the treatment. DXM residues were detected in all samples collected during the treatment, although at low concentration levels, and were not detectable anymore 6 days after treatment suspension [15]. Moreover, a recent study [22] evaluated drug residue levels in urine and feces, as well as its fixation in bovine hair following a single administration of 0.15 mg/kg b.w. dexamethasone acetate and 0.12 mg/kg b.w. dexamethasone sodium phosphate, by different analytical methods based on GC-MS or LC-MS/MS; the results confirmed the high and rapid urinary excretion rate of DXM, with a maximal concentration measured one day after administration and 98% elimination within 3 days [22].

Thus, novel screening tools are needed to accurately reveal the illegal administration of corticosteroids by the meat producing industry.

Our findings demonstrate that low dosages of DXM, administered alone or in association with clenbuterol as growth promoter in beef cattle, according to the protocols often illegally adopted in farm practice [7], induce morphologic changes in the thymus, resulting in increase fat infiltration with concurrent cortical atrophy. The role of thymus atrophy as an indirect biomarker of corticosteroid administration in beef cattle [14,15], as well as in veal calf [7,23] has been previously highlighted. However, the reliability of this method could be affected by several factors, such as infectious diseases, intoxication and stress [24]. In addition, in beef cattle it is rather difficult to distinguish between physiological involution and treatment-induced atrophy, and the thymus score could be influenced by subjective inter-observer variations. Our data indicate that the severity of the histological alteration correlates with the administered DXM dosages, as previously observed [25]. In fact, among animals treated with lower doses of DXM in association with a β-agonist (group C), thymus atrophy was less evident than in group B, and many thymus samples were scored 2. Samples with thymus score 2 are not discriminative, since they occur in both control and treated animals. The association of more objective parameters as indirect indicators may be helpful in enhancing the sensitivity and specificity of the histological method. Administration of synthetic glucocorticoids, such as DXM, can cause acute thymus involution and have been used as a model system [26,27]. Acute thymic involution is characterized by reduction in thymus size caused by acute loss of cortical thymocytes and reduced output of native T cells to the periphery [28]. Differently, age-induced involution is characterized by a gradual expansion of perivascular spaces and reduction of thymic epithelial spaces capable of supporting thymopoiesis [24]. Other possible causes of thymus atrophy as autoimmune phenomenon and immunodeficiency syndromes have been shown to affect cellular density with symmetrical reduction of thymic cortical and medullary areas as well [29]. Our results indicate that the cortex/medulla ratio is a simple and objective parameter, useful for the detection of illegal treatment with low doses of DXM, which could be applied independently from the thymus score. In fact, the C/M value was strongly associated with corticosteroids treatments, with both the protocols applied. The cut off value of 0.93 for the C/M resulted to be highly effective to distinguish control from treated animals with thymus score 2. Furthermore, the C/M value resulted to be not significantly associated with the thymus score. This means that animals with different rates of fat tissue infiltration, due to age-related involution or other factors, could be categorized in treated or untreated group on the basis of the C/M value. In our model, considering 5 lobules randomly selected in each thymus sample, animals with thymus score 2 and at least 2 lobules having C/M < 0.93, should be considered as treated with corticosteroids, with a PPV and NPV both equal to 100%. It should be emphasized that the proposed cut-off value for the C/M has to be validated by studies on a larger population, including different breed and ages of the animals. A similar approach were applied in a recent study on veal calves, treated with low-doses of DXM for 20 days [19]. Results of the study showed that fat infiltration was not significantly associated with steroidal treatment, while a significant reduction in the C/M was observed in treated animals vs controls [19]. The findings of both studies seem indicate that the C/M is strongly related to steroidal treatment in both veal calves and beef cattle, being more reliable than the evaluation of fat infiltration in thymus parenchyma. Assessment of the C/M, once validated in a larger population sample, taking in consideration the repeatability and the accordance between different diagnostic laboratories, could represent a promising and standardizable method for screening purposes, to evaluate the diffusion of corticosteroids illicit treatments in beef production.

In our study, the serum cortisol concentration of control and treated animals was evaluated during the rearing period, as well as at the slaughterhouse. Non treated animals (group A), showed high variability in serum cortisol concentration during the whole study period, with higher median values at T0 and at the slaughterhouse, imputable to physiological stress response to handling and transport procedures [30,31]. The animals treated with DXM showed inhibition of cortisol secretion during the treatment period, as well as at the slaughterhouse, 3 days after treatment suspension. In fact, DXM acts similarly as endogenous cortisol and inhibits hypothalamus and hypophysis activities exerting a negative effect on ACTH secretion [32,33]. These data are in accordance with the findings of a previous experimental study in beef cattle [34], as well as with those of a field study investigating the thymus morphology and serum cortisol concentration in regularly slaughtered beef cattle [14]. In the latter study the animals considered to be negative on the basis of thymus morphology had a significantly higher mean cortisol level than those suspected of corticosteroid treatment [14].

Animals of group C, which were treated with lower doses of DXM in association with clenbuterol, showed inhibition of cortisol secretion during the treatment period, but serum cortisol concentration was restored to physiological levels at slaughterhouse, 8 days after treatment suspension, indicating that the withdrawal period could affect the reliability of the method.

## Conclusions

Our findings support the need for development of new screening methods based on indirect biomarkers, to orientate controls by confirmatory methods. In this regard, the histological evaluation of thymus morphology, and particularly of the C/M may represent a valuable and reproducible method applicable to large-scale screening programs, due to the easy sampling procedures at slaughterhouse, as well as time and cost-sparing of the analysis.

The serum cortisol determination could be considered as an useful *in vivo* biomarker of DXM illegal treatment in beef cattle during the fattening period, whilst it does not appear to be a good biomarker at the slaughterhouse, since the protocol of DXM administration, as well as the withdrawal period could affect the reliability of the method.

Furthermore, the great variability of cortisol baseline values detected in farmed animals at T0, suggests that serum cortisol determination for surveillance policy purpose should be performed on several animals (batch) to account for the inter-individual variability.

## Authors’ contributions

MV led the project, made histological evaluations and drafted the manuscript; KC made statistical analysis and contributed to the manuscript; AS made the determination of serum cortisol concentration and contributed to the manuscript; GB made chemical analysis and contributed to the manuscript; LM contributed to sampling procedures and to the determination of serum cortisol concentration; RS made chemical analysis and contributed to the manuscript; FM supervised the project and contributed to the manuscript. All authors read and approved the final manuscript.

## References

[B1] Renaville R Massart S Lognay G Devolder A Sneyers M Marlier M Severing M Burny A Portetelle D Influence of a hormonal preparation containing glucocorticoids (dexamethasone esters), progestagen (chlormadinone acetate) and oestrogen (ethinyl oestradiol) on testosterone, insulin-like growth factor-1 (IGF-1), IGF-binding proteins and spermatogenic cells in finishing bulls Anim Product 1994 59 189 196 10.1017/S0003356100007674

[B2] Gottardo F Brscic M Pozza G Ossensi C Costiero B Marin A Cozzi G Administration of dexamethasone per os in finishing bulls. I. Effects on productive traits, meat quality and cattle behaviour as indicator of welfare Animal 2008 2 1073 1079 22443708 10.1017/S1751731108002024

[B3] Meyer HH Biochemistry and physiology of anabolic hormones used for improvement of meat production Acta Pathol Microbiol Immunol Scandinavia 2001 109 1 8 10.1111/j.1600-0463.2001.tb00010.x 11297191

[B4] Council Directive 1996 Council Directive 96/23/EC of 29 April 1996 on measures to monitor certain substances and residues thereof in live animals and animal products and repealing Directives 85/358/EEC and 86/469/EEC and Decisions 89/187/EEC and 91/664/EEC. OJ EC L 125:10–31, 23.5

[B5] Commission Regulation 2010 Commission Regulation (EU) N. 37/2010 of 22 December 2009 on pharmacologically active substances and their classification regarding maximum residue limits in foodstuffs of animal origin. OJ EC L 15:1–72, 20.1.2010

[B6] Courtheyn D Le Bizec B Brambilla G De Brabander HF Cobbaert E Van De Wiele Vercammen J De Wasch K Recent developments in the use and abuse of growth promoters Anal Chim Acta 2002 473 71 82 10.1016/S0003-2670(02)00753-5

[B7] Biolatti B Bollo E Cannizzo FT Zancanaro G Tarantola M Dacasto M Cantiello M Carletti M Biolatti PG Barbarino G Effects of low-dose dexamethasone on thymus morphology and immunological parameters in veal calves J Vet Med A 2005 52 202 208 10.1111/j.1439-0442.2005.00714.x 15882406

[B8] Gardini G Del Boccio P Colombatto S Testore G Corpillo D Di Ilio C Urabani A Nebbia C Proteomic investigation in the detection of the illicit treatment of calves with growth-promoting agents Proteomics 2006 6 2813 2822 10.1002/pmic.200500508 16572471

[B9] Toffolatti L Rosa Gastaldo L Patarnello T Romualdi C Merlanti R Montesissa C Poppi L Castagnaro M Bargelloni L Expression analysis of androgen-responsive genes in the prostate of veal calves treated with anabolic hormones Domest Anim Endocrinol 2006 30 38 55 10.1016/j.domaniend.2005.05.008 16023321

[B10] Reiter M Walf VM Christians A Pfaffl MW Meyer HH Modification of mRNA expression after treatment with anabolic agents and the usefulness for gene expression-biomarkers Anal Chim Acta 2007 586 73 81 10.1016/j.aca.2006.10.049 17386698

[B11] Courant F Pinel G Bichon E Monteau F Antignac JP Le Bizec B Development of a metabolomic approach based on liquid chromatography-high resolution mass spectrometry to screen for clenbuterol abuse in calves Analyst 2009 134 1637 1646 10.1039/b901813a 20448932

[B12] Stella R Biancotto G Krogh M Angeletti R Pozza G Sorgato MC James P Andrighetto I Protein expression changes in skeletal muscle in response to growth promoter abuse in beef cattle J Proteome Res 2011 10 2744 2757 10.1021/pr101255c 21425879

[B13] Ministero della Salute 2009 Relazione finale. Piano Nazionale Residui 2009, Roma, Italy 1 63 http://www.salute.gov.it

[B14] Vascellari M Pozza G Poppi L Capello K Angeletti R Ravarotto L Andrighetto I Mutinelli F Evaluation of indirect biomarkers of corticosteroids use as illegal growth promoters in beef cattle Vet Rec 2008 163 147 152 10.1136/vr.163.5.147 18676998

[B15] Cannizzo FT Capra P Divari S Ciccotelli V Biolatti B Vincenti M Effects of low-dose dexamethasone and prednisolone long term administration in beef calf: chemical and morphological investigation Anal Chim Acta 2011 700 95 104 10.1016/j.aca.2010.12.004 21742122

[B16] Council Directive Council Directive 86/609/EEC of 24 November 1986 on the approximation of laws, regulations and administrative provisions of the Member States regarding the protection of animals used for experimental and other scientific purposes. OJ EEC L 358:1–28, 18.12.1986 1986

[B17] Decreto Legislativo 27-1-1992 n 1992

[B18] Commission Decision of 12 August 2002 implementing Council Directive 96/23/EC concerning the performance of analytical methods and the interpretation of results (2002/657/EC). OJ EC L 221:8–36, 17.8.2002 2002

[B19] Bozzetta E Pezzolato M Maurella C Varello K Richelmi GB Draisci R Ferranti C D’Angelo A Caramelli M Development of an enhanced histopathological approach to detect low-dose dexamethasone illicit treatment in veal calves Food Additives and Contaminants A 2011 28 1187 1192 10.1080/19440049.2011.584909 21801077

[B20] Groot MJ Schilt R Ossenkoppele JS Berende PL Haasnoot W Combinations of growth promoters in veal calves: consequences for screening and confirmation methods Zentralblatt für Veterinärmedizin A 2008 45 425 440 10.1111/j.1439-0442.1998.tb00845.x 9793473

[B21] Vincenti M Girolami F Capra P Pazzi M Carletti M Gardini G Nebbia C Study of dexamethasone urinary excretion profile in cattle by LC-MS/MS: comparison between theraputic and growth-promoting administration J Agric Food Chem 2009 57 1299 1306 10.1021/jf803465d 19182943

[B22] Vanhaecke L Antignac JP Courtheyn D Le Bizec B De Brabander H Elimination kinetics of dexamethasone in bovine urine, hair and feces following single administration of dexamethasone acetate and phosphate esters Steroids 2011 76 111 117 10.1016/j.steroids.2010.09.007 20888849

[B23] Cannizzo FT Miniscalco B Riondato F Bollo E Barbarino G Giorgi P Mazzini C Biolatti B Effects of anabolic and therapeutic doses of dexamethasone on thymus morphology and apoptosis in veal calves Vet Rec 2008 163 448 452 10.1136/vr.163.15.448 18849577

[B24] Gruver AL Sempowski GD Cytokines, leptin, and stress-induced thymic atrophy J Leukoc Biol 2008 84 915 923 10.1189/jlb.0108025 18495786 PMC2538595

[B25] Cannizzo FT Spada F Benevelli R Nebbia C Giorgi P Brina N Bollo E Biolatti B Thymus atrophy and regeneration following dexamethasone administration to beef cattle Vet Rec 2010 167 338 343 10.1136/vr.c3303 20802188

[B26] Wyllie AH Glucocorticoid-induced thymocyte apoptosis is associated with endogenous endonuclease activation Nature 1980 284 555 556 10.1038/284555a0 6245367

[B27] Zubkova I Mostowski H Zaitseva M Up-regulation of IL-7, stromal-derived factor-1 alpha, thymus-expressed chemokine, and secondary lymphoid tissue chemokine gene expression in the stromal cells in response to thymocyte depletion: implication for thymus reconstitution J Immunol 2005 175 2321 2330 16081802 10.4049/jimmunol.175.4.2321

[B28] Wang SD Huang KJ Lin YS Lei HY Sepsis-induced apoptosis of the thymocytes in mice J Immunol 1994 152 5014 5021 8176219

[B29] Valli VEO Maxie MG Hematopoietic system Jubb, Kennedy, and Palmer’s Pathology of domestic animals 2007 5 Elsevier, Amsterdam 107 324

[B30] Grandin T Assessment of stress during handling and transport J Anim Sci 1997 75 249 257 9027573 10.2527/1997.751249x

[B31] Odore R D’angelo A Badino P Bellino C Pagliasso S Re G Road transportation affects blood hormone levels and lymphocyte glucocorticoid and β adrenergic receptor concentrations in calves Vet J 2004 168 297 303 10.1016/j.tvjl.2003.09.008 15501147

[B32] Meij BP Mol JA Kaneko JJ, Harvey JW, Bruss ML Adrenocortical function Clinical biochemistry of domestic animals 2008 6 Academic, San Diego 605 622

[B33] Abraham G Allersmeier M Gottschalk J Schusser GF Hoppen HO Ungemach FR Effects of dermal dexamethasone application on ACTH and both basal and ACTH-stimulated cortisol concentration in normal horses J Vet Pharmacol Theriol 2009 32 379 387 10.1111/j.1365-2885.2008.01054.x 19614843

[B34] Marin A Pozza G Gottardo F Moro L Stefani AL Cozzi G Brscic M Andrighetto I Ravarotto L Administration of dexamethasone per os in finishing bulls: II. Effects on blood parameters used as indicators of animal welfare Animal 2008 2 1080 1086 22443709 10.1017/S1751731108002061

